# Impact of pre-operative breast magnetic resonance imaging on contralateral synchronous and metachronous breast cancer detection—A case control comparison study with 1468 primary operable breast cancer patients with mean follow-up of 102 months

**DOI:** 10.1371/journal.pone.0260093

**Published:** 2021-11-18

**Authors:** Wen-Pei Wu, Chih-Yu Chen, Chih-Wei Lee, Hwa-Koon Wu, Shou-Tung Chen, Yu-Ting Wu, Ying-Jen Lin, Dar-Ren Chen, Shou-Jen Kuo, Hung-Wen Lai

**Affiliations:** 1 Department of Medical Imaging, Changhua Christian Hospital, Changhua, Taiwan; 2 Department of Biomedical Imaging and Radiological Sciences, National Yang Ming University, Taipei, Taiwan; 3 Kaohsiung Medical University, Kaohsiung, Taiwan; 4 Division of Medical Imaging, Yuanlin Christian Hospital, Yuanlin, Taiwan; 5 Endoscopic & Oncoplastic Breast Surgery Center, Department of Surgery, Changhua Christian Hospital, Changhua, Taiwan; 6 Division of General Surgery, Department of Surgery, Changhua Christian Hospital, Changhua, Taiwan; 7 Department of Surgery, Wuri Lin Shin Hospital, Taichung, Taiwan; 8 Tumor Center, Changhua Christian Hospital, Changhua, Taiwan; 9 Comprehensive Breast Cancer Center, Department of Surgery, Changhua Christian Hospital, Changhua, Taiwan; 10 Minimal Invasive Surgery Research Center, Changhua Christian Hospital, Changhua, Taiwan; 11 School of Medicine, National Yang Ming University, Taipei, Taiwan; 12 School of Medicine, Chung Shan Medical University, Taichung, Taiwan; 13 Division of Breast Surgery, Yuanlin Christian Hospital, Yuanlin, Taiwan; 14 Chang Gung University College of Medicine, Taoyuan City, Taiwan; 15 Division of General Surgery, Kaohsiung Chang Gung Memorial Hospital, Kaohsiung, Taiwan; Medical University of Vienna, AUSTRIA

## Abstract

**Background:**

Women with unilateral breast cancer are at an increased risk for the development of contralateral breast cancers. We hypothesis that combined breast MRI would detect more contralateral synchronous breast cancer than conventional imaging alone, and resulted in less contralateral metachronous breast cancer during follow-up.

**Methods:**

We retrospectively collected two groups of breast cancer patients diagnosed from 2009 to 2013 for evaluating the effectiveness and value of adding pre-operative breast MRI to conventional breast images (mammography and sonography) for detection of contralateral synchronous breast cancer. The new metachronous contralateral breast cancer diagnosed during follow-up was prospectively evaluated and compared.

**Results:**

Group A (n = 733) comprised patients who underwent conventional preoperative imaging and group B (n = 735) combined with MRI were enrolled and compared. Seventy (9.5%) of the group B patients were found to have contralateral lesions detected by breast MRI, and 65.7% of these lesions only visible with MRI. The positive predictive value of breast MRI detected contralateral lesions was 48.8%. With the addition of breast MRI to conventional imaging studies, more surgical excisions were performed in contralateral breasts (6% (44/735) versus 1.4% (10/733), P< 0.01), more synchronous contralateral breast cancer detected (2.9% (21/735) versus 1.1% (8/733), P = 0.02), and resulted in numerical less (2.2% (16/714) versus 3% (22/725), p = 0.3) metachronous contralateral breast cancer during a mean follow-up of 102 months.

**Conclusions:**

Our study provides useful estimates of the pre-operative breast MRI for the increased detection of contralateral synchronous breast cancer and less subsequent contralateral metachronous breast cancer.

## Background

Women with unilateral breast cancer are at an increased risk for the development of contralateral breast cancers, with a 1%–5% incidence of synchronous cancer and a 3%–13% incidence of metachronous cancer [1–4]. Moreover, women with bilateral breast cancer were found to have worse prognoses than those with unilateral breast cancer [5–7]. Methods to enhance early detection of contralateral synchronous breast cancer and/or decrease metachronous contralateral breast cancer would be important for newly diagnosed patients.

Contrast-enhanced dynamic magnetic resonance imaging (MRI) has been shown to be a useful imaging modality for the diagnosis of breast cancer [8,9], estimating tumor size [10,11], and detecting occult breast lesions [2,3,12]. Some studies reported that breast MRI can detect 3–5% of occult tumors in the contralateral breast [1–4,13–27]. However, case control comparison study for evaluation of adding breast MRI to conventional breast imaging for contralateral synchronous breast cancer detection, and the impact of pre-operative MRI to the subsequent occurrence of metachronous contralateral breast cancer was rarely reported [21,24].

We hypothesis that the high sensitivity of breast MRI would detect more synchronous contralateral breast cancer than conventional imaging group, and resulted in less metachronous contralateral breast cancer found during follow-up. To confirm our hypothesis, we conducted a case control comparison study to evaluate the diagnostic performance of adding pre-operative breast MRI to conventional breast imaging in the detection of synchronous contralateral breast cancer for women with primary operable breast cancer. The incidence, pathology, and management of patients with MRI detected contra-lateral occult breast lesions, impact of ipsilateral and contralateral breast surgery, and new metachronous contralateral breast cancer diagnosed during follow-up were analyzed and reported.

## Methods

### Patients

In this case control comparison analysis, we retrospectively collected two groups of patients for evaluating the effect and value of adding pre-operative breast MRI to conventional breast images (mammography and sonography) for detection of contralateral synchronous breast cancer. A retrospective review of patients who underwent operations for breast cancer from January 2009 to December 2013 was conducted at Changhua Christian Hospital (CCH), a tertiary medical center at central Taiwan. Patients, who diagnosed as primary operable breast cancer, received pre-operative evaluation with conventional breast imaging combined with or without MRI, and received definite breast cancer operations at CCH were included. Patients who did not receive surgery because of distant metastasis or neoadjuvant chemotherapy (n = 148) were excluded. Patients who had discrepancy in clinical examination, mammography and ultrasound, suspicion of multifocal or multicentric disease, invasive lobular carcinoma, or who breast-conserving therapy is planned received pre-operative breast MRI [28].

The type of operation (breast-conserving surgery (BCS), mastectomy or mastectomy with breast reconstruction), and contralateral breast surgeries were compared. The MRI images and reports were reviewed to identify whether contralateral breast lesions were detected. The subsequent biopsy results and surgical methods were reviewed. To evaluate the impact of different pre-operative imaging methods on contralateral metachronous breast cancer occurrence, patients were prospectively followed up. During the follow-up examinations, patients were advised to undergo annual mammography and bilateral whole-breast sonography every 6 months for the first 2 years and annual mammographic and sonographic evaluations thereafter. Total incidence of recurrence or death due to breast cancer were ascertained at the most recent follow-up, which ended on 30 Sep 2020. The clinicopathologic and imaging data collection was performed by special trained study nurse (SHP), and verified by principle investigator (HWL). This study was approved by the institutional review board (IRB) of CCH (CCH IRB No.140404). Informed consent was waived because of the retrospective nature of this study and the analysis used anonymous clinical data.

### Diagnostic imaging equipment/conventional breast imaging

Patients who received mammography were imaged using a Hologic Lorad Selenia Digital Mammography machine. Standard mediolateral oblique (MLO) and craniocaudal (CC) views of mammograms were obtained for all patients. For automatic volumetric measurement, all mammograms were processed with Volpara software (v.1.5.2.0, Volpara Health Technologies, Wellington, New Zealand) to obtain breast density grades. The four density grades correspond to grades a, b, c, and d of the fifth edition BI-RADS classification. The breast densities were then reclassified as fatty breast (including grades a and b), and dense breast (including grades c and d). Ultrasound procedures were performed with the patient in the supine position. Imaging was performed with a high-resolution 5–12 MHz linear array transducer, including color Doppler ultrasonography (Voluson 530D and 730D). The sonography examinations were carried out by experienced, board-certified breast physicians.

### MR imaging of study

The MRI protocol used in current study was reported in previous studies [11,29,30] and summarized. MR imaging was performed with a Siemens (Verio) 3.0 Tesla magnet. All patients were imaged in the prone position with both breasts placed into a dedicated 16 channel breast coil. The whole breast MRI readings were carried out by experienced, board-certified breast radiologist (HKW). All the breast imagines, including mammography, sonography and MRI, were recorded according to the American College of Radiology Breast Image Reporting and Data System (BI-RADS) [31].

### Statistical analyses

Data are expressed as mean ± standard deviation (SD) for continuous variables. Independent *t* tests were used for the comparison of continuous variables. Categorical variables were normally tested by the χ2 test when appropriate. All *p* values are two-tailed; a *p* value of less than 0.05 was considered to indicate statistical significance. All statistical analyses were performed with SPSS 19.0 software (IBM).

## Results

**Fig 1:**
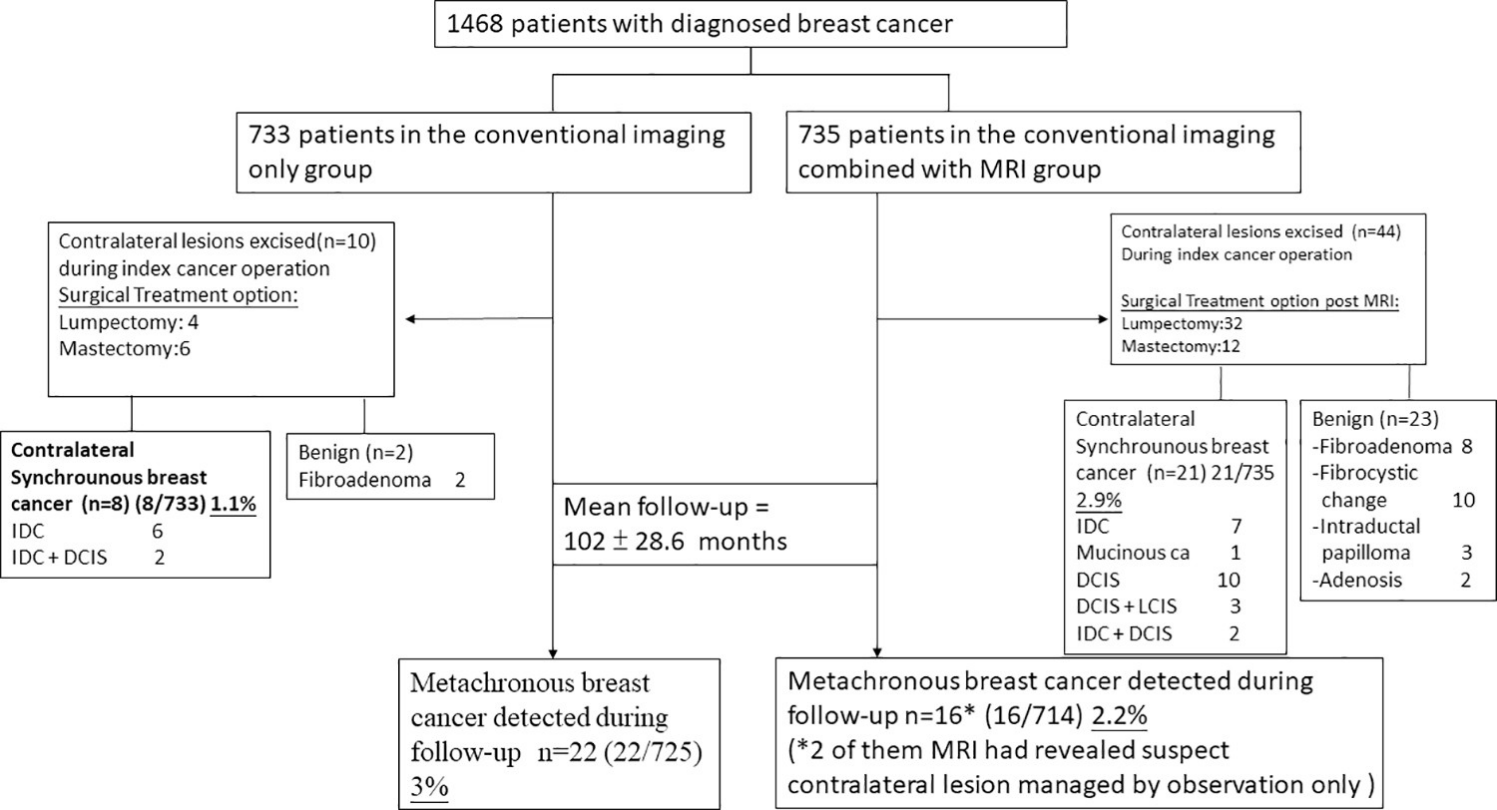
The flowchart presented the current case control comparison study, which enrolled a total of 1468 patients. Group A (n = 733) comprised patients who underwent conventional preoperative imaging (mammography and sonography) and group B (n = 735) comprised patients who received MRI combined with conventional imaging. With the addition of breast MRI to conventional imaging studies, more surgical excisions were performed in contralateral breasts (6% (44/735) versus 1.4% (10/733), P< 0.01), more synchronous contralateral breast cancer detected (2.9% (21/735) versus 1.1% (8/733), P = 0.02).

A total of 1468 patients fulfilled the inclusion criteria and were enrolled in this study. Patients were stratified into two preoperative imaging groups. Group A (n = 733) comprised patients who underwent conventional preoperative imaging (mammography and sonography) and group B (n = 735) comprised patients who received MRI combined with conventional imaging (Fig 1 shows the flow chart of patients’ management in the current study). In current case control comparison study, there were no significant clinicopathologic differences between patients who received conventional preoperative imaging alone (group A, 733 patients) and those who underwent preoperative MRI (group B, 735 patients) in addition to conventional imaging (Table 1).

**Table 1:** Clinicopathologic characteristics of patients and tumors in current case control comparison study.

		No MRI group (n = 733) n(%)	MRI group (n = 735) n(%)	P value
Age (years)		52.15±11.39	52.73±10.74	0.313
Breast density on mammography	Fatty breast	119 (16.2)	104 (14.2)	0.266
	Dense breast	614 (83.8)	631 (85.8)	
Location of index tumor	Right	363 (49.5)	341 (46.4)	0.230
	Left	370 (50.5)	394 (53.6)	
Biopsy method	CNB	566 (78.9)	634 (87.1)	<0.001
	Stereotactic biopsy	61 (8.5)	83 (11.4)	
	Excision	82 (11.4)	5 (0.7)	
	FNAC	8 (1.1)	6 (0.8)	
Tumor size (cm)		2.15±1.64	2.27±1.62	0.165
Lymph node	Positive	242 (33.0)	241 (33.5)	0.839
	Negative	491(67.0)	478 (66.5)	
Stage	O	120 (16.6)	117 (15.9)	0.287
	I	219 (30.2)	240 (32.7)	
	II	284 (39.2)	302 (41.1)	
	III	97 (13.4)	73 (9.9)	
	IV	4 (0.6)	3 (0.4)	
Grade	I	128 (18.5)	118 (16.9)	<0.001
	II	421 (60.8)	364 (52.0)	
	III	144 (20.8)	218 (31.1)	
ER	Positive	538 (75.0)	576 (79.4)	0.046
	Negative	179 (25.0)	149 (20.6)	
PR	Positive	533 (74.3)	535 (73.8)	0.814
	Negative	184 (25.7)	190 (26.2)	
HER-2	Positive	162 (23.2)	160 (22.9)	0.887
	Negative	535 (76.8)	538 (77.1)	

Mean ± standard deviation (S.D.), CNB: Core needle biopsy, FNAC: Fine needle aspiration cytology, ER: Estrogen receptor, PR: Progesterone receptor, HER-2: Human epidermal growth factor receptor-2.

In the group B, 70 (9.5%) patients were found to have contralateral breast lesions detected by breast MRI, and 65.7% (46/70) of the lesions were only visible with MRI (Table 2). Among the 70 patients, 3 (4.3%) patients had BI-RADS category 2, 20 (28.6%) patients had BI-RADS category 3, 41 (58.6%) patients had BI-RADS category 4, and 6 (8.6%) patients had BI-RADS category 5. All malignancies were confirmed via surgical excisions in patients who had BI-RADS category 5. Among 41 patients with BI-RADS category 4 MRI-detected lesions, 37 patients received surgical excisions and 15(40.5%) were found to have malignancy. Only 1 patient with BI-RADS category 3 received pathologic check-up and was found to have a benign lesion. The positive predictive value of BI-RADS category 4 or 5 in our study is 48.8% (21/43).

**Table 2:** MRI detected contralateral lesions correlated to conventional imaging, BI-RADS category, and pathologic results.

	MRI-detected lesions (n = 70)			
	Correlated with conventional images (n = 24)		Not correlated with conventional images (n = 46)	
		BI-RADS Category		BI-RADS Category
Received Operation (n = 44)	18 (75%)	Category 3: 1 Category 4: 14 Category 5: 3	26 (56.5%)	Category 4: 23 Category 5: 3
Benign (n = 23)	n = 10	Category 3: 1 Category 4: 9	n = 13	Category 4: 13
Malignant (n = 21)	n = 8	Category 4: 5 Category 5: 3	n = 13	Category 4: 10 Category 5: 3
Not Received Operation (n = 26)	6 (25%)	Category 3: 5 Category 4: 1	20 (43.5%)	Category 2: 3 Category 3: 14 Category 4: 3

Data are number of lesions. BI-RADS = Breast Imaging Reporting and Data System.

In conventional imaging group, 10 patients received contralateral breast operations, and 8 of them were found to have malignant lesion in the final pathology check-up (Fig 1). While Forty-four patients of combined MRI group received contralateral breast operations, and at pathologic check-up, 21 were with malignancy, and 23 were benign lesions. Higher synchronous contralateral breast cancer detection rate was observed in the cohort of combined MR imaging group (2.9% versus 1.1%, P = 0.02).

Among these 21 detected occult contralateral breast cancers in 735 patients of Group B, 8 (1.1%) were both detected by conventional imaging and MRI, and 13 (1.8%) were only detected by MRI (Table 2). Among these lesions, 10(47.6%) were DCIS, 3(14.3%) DCIS with lobular carcinoma in situ, 7(33.3%) invasive ductal carcinoma, and 1(4.8%) mucinous carcinoma (Fig 1). Compared with the pathology of the 735 primary operable breast cancers (group B), the MRI detected synchronous contra-lateral breast cancer were associated with higher incidence of in situ carcinoma (61.9% versus 15.9%, P<0.01).

**Fig 2:**
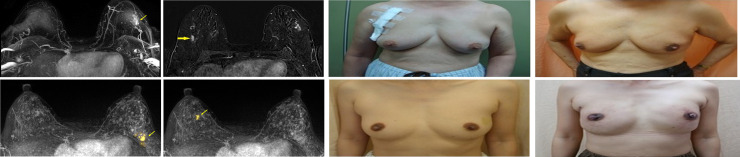
(a-h) Case A: A 75-year-old patient was mammography diagnosed with left breast tumor primarily but the contralateral lesion was found via MR Imaging. She received a bilateral endoscopy assisted partial mastectomy. The final pathology proved the infiltrating ductal carcinoma (IDC) in the right. (e-h) Case B: A 49-year-old patient with pathologic diagnosed of ductal carcinoma in situ (DCIS) of left breast. The contralateral lesion was found via MRI before operation. According to her family history of breast cancer, she received an endoscopic assisted bilateral nipple-sparing mastectomy with immediate breast reconstruction. The final pathology proved bilateral DICS.

There were no differences in surgical methods (BCS vs mastectomy) employed between the two groups of patients (P = 0.13, Fig 2) for the initial primary diagnosed breast cancer. Six (0.8%) of patients received bilateral mastectomy in the conventional imaging group, and 12 (1.6%) in the combined MRI group received bilateral mastectomy (P = 0.24). Sixty-one (17.1%) patients in conventional imaging group received breast reconstructions, while 154 (39.8%) patients in combined with MRI group received breast reconstructions (P<0.01).

**Fig 3:**
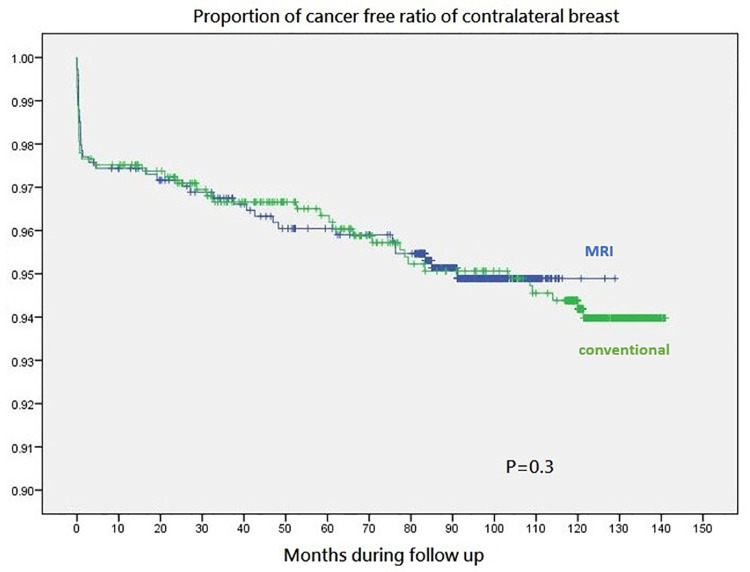
During a median follow-up of 102±28.6 months, metachronous contralateral breast cancers were found in 22 patients at conventional imaging group, and 16 patients at combined with MRI group. There were numerical less patients of new metachronous breast cancer in patients received pre-operative combined imaging with MRI compared to conventional imaging alone (2.2% (16/714) versus 3% (22/725), p = 0.3).

During a mean follow-up of 102 ± 28.6 months, metachronous contralateral breast cancers were found in 22 patients at conventional imaging group, and 16 patients at combined with MRI group (Fig 3). Two of these 16 breast cancer patients had initial MRI suspect contralateral breast lesions and decided to receive conservative follow-up. There was a numerical less patients of new metachronous contralateral breast cancer in patients received pre-operative combined imaging with MRI compared to conventional imaging alone (2.2% (16/714) versus 3% (22/725), p = 0.3). The contralateral breast cancer detection by MRI in current study was summarized and compared with literature reviews in Table 3.

**Table 3:** Published studies documenting pre-operative MRI Evaluation of the contralateral breast in women with newly diagnosed breast cancer.

First author	Year	Journal	No. of Patients	MRI positive	True positive	False positive	PPV	Cancer Detection Rate
Fischer^4^	1999	Radiology	332	30	15	15	50%	4.5%
Liberman^15^	2003	AJR	212	61	12	49	19.7%	5.7%
Hollingsworth^16^	2006	J Okla State Med Assoc	334	NA	NA	NA	NA	3.6%
Lehman^1^	2007	N Eng J Med	969	135	30	91	24.8%	3.1%
Renz^17^	2010	Breast Cancer Res Treat	875	NA	42	NA	NA	4.8%
Berg^18^	2012	AJR	367	54	14	40	28%	3.8%
Taneja^19^	2012	Indian J Radiol Imaging.	294	25	16	9	64%	4.1%
Kim^20^	2012	J Ultrasound Med	853	98	17	81	17.3%	2.0%
Fan^28^	2013	Breast J	445	NA	22	NA	NA	4.9%
Butler^29^	2013	World J Radiol	234	127	47	80	37.0%	20.1%
Bae^30^	2013	AJR	308	45	24	21	53%	8%
Kim^21^	2013	Radiology	1771 (MRI) 1323 (No MRI)	49 NA	25 18	24 NA	51% NA	2.6% (1.4%+1.2%) 1.4%
Gonzalex^22^	2014	World J Surg	440	24	4	20	16.7%	0.9%
Barco^23^	2016	Eur J Radiol.	1513	NA	26	NA	NA	1.7%
Wang^24^	2016	Journal of Clinical Oncology	6377 (MRI) 12754 (No MRI)	NA NA	375 263	NA NA	NA NA	5.9% 2.1%
Elder^25^	2017	Ann Surg Oncol.	683	108	8	100	7.4%	1.2%
Jonna^26^	2017	Breast Cancer Res Treat	435	29	15	14	51.7%	3.4%
Santiago^27^	2018	Curr Probl Diagn Radiol.	311	NA	15	NA	NA	4.8%
Susnik^13^	2018	Journal of Surgical Oncology	1894	201	60	141	29.9%	3.2%
Raghavendra^14^	2019	Breast Cancer Res Treat	1116	118	20	98	16.9%	1.8%
Wu (current)			735 (MRI) 733 (No MRI)	44 NA	21 8	23 NA	48.8% NA	2.9% (1.1%+1.8%) 1.1%

Note: The results of the studies which enrolled more than 200 patients were summarized in this table. PPV: Positive predictive value, NA: Not available, MRI: Magnetic resonance imaging.

## Discussion

To evaluate the efficacy of combined breast MRI in the detection of synchronous contralateral breast cancer and the impact of consequent metachronous contralateral breast cancer, we performed a case control comparison study, which enrolled 1468 primary operable breast cancer patients with two different groups of pre-operative breast imaging modalities and received surgery at a single institution, with a mean follow-up of 102 ± 28.6 months. We found that, with the addition of breast MRI to conventional imaging studies, more surgical excisions were performed in contralateral breasts, more synchronous contralateral breast cancer detected, and resulted in numerical less metachronous contralateral breast cancer during follow-up.

In Brennan et al.’s study for evaluation of the contralateral breast cancer detection by pre-operative MRI, the incidence of suspicious MR imaging findings was 9.3%, with a PPV 47.9%, and a false-positive rate of 52% [32]. In our current study, MR imaging detected 70(9.5%) contralateral lesions in 735 patients, and 21 of them were proved to be malignancy. The PPV of the MRI detected contralateral lesions in our study was 48.8%, which is compatible to literature reported series (Table 3).

As showed in Fig 1, considering the incidence of synchronous contralateral cancers (2.9% vs 1.1%) and of metachronous contralateral cancers (2.2% vs 3%) in the contralateral MR imaging–screened and comparison groups. Therefore, we hypothesize that pre-operative breast MRI can earlier detect occult contralateral cancers, which reduces subsequent metachronous cancers during 102 months.

The detection of additional abnormal enhancing lesions in breast MRI might be “problematic” for subsequent patient management. The positive predictive value of MRI-detected suspicious lesions are 48.8% in our study. Second-look ultrasound and image-guided core needle biopsy for suspicious lesion could obtain tissue diagnosis and decrease unnecessary operations [33]. Second-look ultrasound is preferable in the clinical setting, whenever possible, as ultrasound is well-tolerated, cost-effective, and time-saving for patients and surgeons. However, using ultrasound or mammography to guide the treatment of MRI-detected lesions sometimes encounters the issue of lesion matching. In our study, 65.7% (46/70) lesions cannot correlate with mammography or ultrasound.

Controversies existed that MRI was reported to have increase ipsilateral mastectomy rate and bilateral mastectomy rate [34]. In current case control analysis, the mastectomy rate in the combined MRI cohort of patients was not significantly increased when compared with the conventional imaging cohort (52.7% versus 48.6%, P = 0.13). The higher number of patients received bilateral mastectomy in combined MRI group were in part due to higher synchronous contralateral breast cancer detection before operation. However, a substantial of patients received bilateral mastectomy due to psychologic stress of the suspicious lesions detected by breast MRI. We also observe a numerical but not statistically significant increase of bilateral mastectomy cases in patients received pre-operative evaluation with MRI than conventional imaging only group (12 out of 735 versus 6 out of 733, P = 0.24). A significantly increase of breast reconstructions (39.8% versus 17.1%, P<0.01) was observed in patients received combined MRI evaluation than conventional breast imaging alone. Other study had suggested that pre-operative MRI study may increase the mastectomy rate and therefore increased the breast reconstruction rate [35,36].

In our total 1468 breast cancer patients, 29 (2%) synchronous contralateral breast cancer was detected. Most (58.6%, 17/29) of them were DCIS lesions. Compared with the pathology of the primary index breast cancer, either in the combined MRI group (61.9% versus 15.9%, P<0.01) or in the conventional breast imaging cohort (50% versus 16.6%), the detected synchronous contralateral breast cancers were associated with higher in situ carcinoma. These findings could explain why synchronous contralateral breast cancer, which usually presented in an earlier stage than the primary breast cancer [13], was a challenge for clinicians for early detection.

During a mean follow-up of 102 ± 28.6 months, we observed 38 patients developed new metachronous contralateral breast cancer, and 22 (3%) of them were in initial conventional pre-operative imaging group, and 16(2.2%) of them in combined MRI group (Fig 3). Contralateral metachronous cancer could be either synchronous occult cancers not detected by pre-operative imaging and diagnosed during follow-up or could be new contralateral breast cancer developed after initial primary index breast cancer operations. The higher rate of contralateral synchronous breast cancer detected and lower number of contralateral metachronous breast cancer found during follow-up in the pre-operative combined with MRI group (Figs 1 and 3) supported the hypothesis that combined with pre-operative MRI could detected more synchronous breast cancer than conventional imaging alone, and during follow-up some occult lesions not detected in contralateral breast at time of primary index cancer operations would be diagnosed as metachronous breast cancer.

Our study was limited due to its retrospective nature and possible selection bias. Our study does not look at recurrence rates or local recurrence-free survival rates between MRI and no-MRI groups or in distant recurrence rates. In addition, even though 4 patients with BI-RADS category 4 in our study did not receive pathologic confirmation in our study, but no malignancies were confirmed during the follow up. Patients with contralateral occult lesions detected by conventional imaging alone or combined with MRI did not receive surgical excision in every case to confirm the nature of the lesion. This might, therefore, underestimate the actual synchronous breast cancer rate. However, we did collect 733 primary operable breast cancer patients with pre-operative conventional breast imaging, and 735 combined MRI patients who received surgical intervention at a single institution with detailed pathologic report and with a mean follow-up of 102 ± 28.6 months, which enable us to complete this case control comparison study with the occurrence of new metachronous contralateral breast cancer. We provide solid evidence that adding breast MRI increase the contralateral occult synchronous breast cancer detection rate from 1.1% to 2.9%, and adding MRI increased about 1.8% of contralateral occult breast cancer detection rate (Table 2).

## Conclusions

In conclusion, in current case control comparison study, we found an increase of contralateral synchronous breast cancer detection during primary index breast cancer operation and numerical lower number of metachronous contralateral breast cancer occurrence during follow-up in patients with primary operable breast cancer received pre-operative combined MRI evaluation than conventional breast imaging alone. The risk of synchronous contralateral breast cancer should be kept in mind for pre-operative evaluation of primary operable breast cancer patients. Biopsy procedure for tissue diagnosis or surgical strategy discussed with patients is warranted if suspicious synchronous contralateral lesion been detected by conventional imaging and/or breast MRI.

## Supporting information

S1 Data (XLSX)
